# Incremental Prognostic Value of Pericoronary Adipose Tissue Thickness Measured Using Cardiac Magnetic Resonance Imaging After Revascularization in Patients With ST-Elevation Myocardial Infarction

**DOI:** 10.3389/fcvm.2022.781402

**Published:** 2022-03-04

**Authors:** Yue Ma, Quanmei Ma, Xiaonan Wang, Tongtong Yu, Yuxue Dang, Jin Shang, Guangxiao Li, Yang Hou

**Affiliations:** ^1^Department of Radiology, Shengjing Hospital of China Medical University, Shenyang, China; ^2^Department of Cardiology, Shengjing Hospital of China Medical University, Shenyang, China; ^3^Department of Medical Record Management Center, The First Hospital of China Medical University, Shenyang, China

**Keywords:** ST elevated myocardial infarction, magnetic resonance imaging, pericoronary adipose tissue, coronary artery disease, prognosis

## Abstract

**Background and Aim:**

Pericoronary adipose tissue (PCAT) reflects pericoronary inflammation and is associated with coronary artery disease. We aimed to identify the association between local PCTA thickness using cardiac magnetic resonance (CMR) and prognosis of patients with ST-elevation myocardial infarction (STEMI), and to investigate the incremental prognostic value of PCAT thickness in STEMI after reperfusion.

**Methods:**

A total of 245 patients with STEMI (mean age, 55.61 ± 10.52 years) who underwent CMR imaging within 1 week of percutaneous coronary intervention therapy and 35 matched controls (mean age, 53.89 ± 9.45 years) were enrolled. PCAT thickness indexed to body surface area at five locations, ventricular volume and function, infarct-related parameters, and global strain indices were evaluated using CMR. Associations between PCAT thickness index and 1-year major adverse cardiovascular events (MACE) after STEMI were calculated. The prognostic value of the standard model based on features of clinical and CMR and updated model including PACT thickness index were further assessed.

**Results:**

Patients with MACE had a more significant increase in PCAT thickness index at superior interventricular groove (SIVGi) than patients without MACE. The SIVGi was significantly associated with left ventricular ejection fraction (LVEF), infarct size, and global deformation. SIVGi > 4.98 mm/m^2^ was an independent predictor of MACE (hazard ratio, 3.2; 95% CI: 1.6–6.38; *p* < 0.001). The updated model significantly improved the power of prediction and had better discrimination ability than that of the standard model for predicting 1-year MACE (areas under the ROC curve [*AUC*] = 0.8 [95% *CI*: 0.74–0.87] *vs*. *AUC* = 0.76 [95% *CI*: 0.68–0.83], *p* < 0.05; category-free net reclassification index [*cfNRI*] = 0.38 [95% *CI*: 0.1–0.53, *p* = 0.01]; integrated discrimination improvement [*IDI*] = 0.09 [95% *CI*: 0.01–0.18, *p* = 0.02]).

**Conclusions:**

This study demonstrated SIVGi as an independent predictor conferred incremental value over standard model based on clinical and CMR factors in 1-year MACE predictions for STEMI.

## Introduction

Effective stratification of residual risk during the early postoperative period is particularly important for the short-term prognosis of patients with acute myocardial infarction (MI) (1). Previous studies have shown that decreased cardiac function, morphological changes of the left ventricle, and large myocardial infarct size evaluated using cardiac magnetic resonance (CMR) imaging are common predictors of adverse events after MI (2). CMR strain indicators, which signal decreased myocardial compliance, have become important prognostic and functional markers for early risk stratification and treatment decisions for survivors of MI (3, 4).

Inflammation has long been postulated to play an important role in the atherosclerotic progression and recurrent major adverse cardiovascular events (MACE) (5, 6). Reportedly, the volume and thickness of epicardial adipose tissue (EAT), as a source of inflammatory cytokines, is associated with the severity of coronary artery disease (CAD) (7–9). Recent evidence demonstrates that the CT attenuation of pericoronary adipose tissue (PCAT) – the EAT directly surrounding the coronary arteries – is associated with cardiac mortality (10, 11). It might imply that the local PCAT might have a more direct and efficient impacts on CAD compared with the global EAT. However, there are only a few studies on the relationship between local PCAT and ST-elevation myocardial infarction (STEMI), and no study has been reported on the incremental prognostic value of PCAT thickness after revascularization in patients with STEMI.

Therefore, the purpose of this study was to evaluate the changes in PCAT thickness at different locations after STEMI, to evaluate the association of PCAT thickness indices with the occurrence MACE, and to determine the effective predictors among PCAT thickness indices to investigate the incremental prognostic value of PCAT thickness index in MACE within 1 year after STEMI.

## Materials and Methods

### Study Group

From April 2017 to December 2019, we retrospectively screened 323 patients who had STEMI for the first time and underwent percutaneous coronary intervention (PCI) therapy at Shengjing Hospital of China Medical University. STEMI was defined according to current definitions (12). We excluded patients who did not undergo contrast CMR imaging within 1 week of PCI, had CMR images of poor quality, or had a history of MI or revascularization. A total of 245 patients met the eligibility criteria and were enrolled in the study (Supplementary Figure 1). We also enrolled 35 participants without any known or suspected CAD (based on clinical history) who underwent CMR imaging between December 2019 and July 2020 as control subjects. This study complied with the tenets of the Declaration of Helsinki and was approved by the Shengjing Hospital of China Medical University Research Ethics Committee.

### Clinical Assessment and Endpoint

We collected the relevant clinical history data of all patients and controls, including information on baseline clinical characteristics, cardiovascular risk factors, laboratory parameters, current medication, and angiographic information. The follow-up information was obtained from the patients' medical records and from telephone interviews. The endpoint was time to first MACE within the 12-month follow-up period post-revascularization, and included a composite of cardiovascular death, recurrent MI, clinically driven target lesion revascularization, and readmission for heart failure (13). Planned-staged procedures were not counted as MACE.

### CMR Imaging and Analysis

All patients were examined using a 3.0-T MR scanner (Ingenia, Philips Healthcare, Best, Netherlands). Anonymized CMR images were analyzed offline using customized software (Circle Cardiovascular Imaging, cvi42^®^, Calgary, Canada). The images were analyzed by two experienced observers blinded to all patient data. CMR data were prospectively incorporated into the database. The details of the technical aspects of CMR acquisitions, sequences, and quantification have been described previously (14).

Left ventricular ejection fraction (LVEF) (%), left ventricular end-diastolic volume (LVEDV) index (ml/m^2^), left ventricular end-systolic volume (LVESV) index (ml/m^2^), left ventricular mass index, right ventricular ejection fraction (%), right ventricular end-diastolic volume index (ml/m^2^), right ventricular end-systolic volume index (ml/m^2^), microvascular obstruction (%), edema size (% of left ventricular mass), infarct size (% of left ventricular mass), and myocardial salvage index (% of left ventricular mass with myocardial edema not showing delayed enhancement) were calculated. Tissue tracking-CMR strain analysis was also performed, and the global strain values (global radial strain [GRS], global circumferential strain [GCS], and global longitudinal strain [GLS]) through the entire cardiac cycle were calculated semi-automatically using the software as previously described (14).

### Measurement of PCAT Thickness

Pericoronary adipose tissue thickness was measured using the end-diastolic phase CMR cine images (Supplementary Figure 2). PCAT thickness was defined as the distance between the myocardial surface and the pericardium, which is perpendicular to the pericardium through the center of coronary arteries inside the PCAT. All PCAT measurements were indexed to the body surface area of individual patients to get corrected parameters (right atrioventricular groove PCAT thickness index [RAVGi], anterior interventricular groove PCAT thickness index [AIVGi], left atrioventricular groove PCAT thickness index [LAVGi], superior interventricular groove PCAT thickness index [SIVGi], inferior interventricular groove PCAT thickness index [IIVGi]).

### Statistical Analysis

Continuous data were tested for normal distribution using the Kolmogorov–Smirnov test. Normally distributed data were expressed as mean ± SD and compared using Student's *t*-test. Nonparametric data were expressed as medians (interquartile ranges) and compared using the Mann–Whitney *U* test. Categorical variables were expressed as percentages and compared using the chi-square test or Fisher's exact test where appropriate. The correlations between PCAT thickness indices and CMR imaging parameters were evaluated using Pearson's correlation coefficient and the reproducibility was assessed using Bland–Altman analysis and intra-class correlation coefficients.

The time-dependent receiver operating characteristics (ROC) curve analysis was used to assess the performance of the strain indexes and PCAT thickness indexes for the prediction of adverse cardiac events 1-year post STEMI. The areas under the ROC curve (AUCs) and the corresponding 95% *CI* were calculated as well. Meanwhile, the optimal cutoff for each index was determined. Kaplan–Meier curves were used to estimate MACE-free survival rates with respect to high and low level of the PCAT index with best discriminative ability. The comparison of survival rates between groups was examined using the log-rank test.

To facilitate the construction of the Cox regression model, continuous variables were categorized into binary variables using optimal cutoffs, median values, or values of clinical significance, as appropriate. Univariate Cox regression model was used to estimate the risk of adverse events, which were expressed as hazard ratios (*HR*) and 95% *CIs*. Two multivariable Cox regression models were then conducted. Those were the standard model and updated model. The least absolute shrinkage and selection operator (LASSO) strategy, an efficient in handling high-dimensional data (15), was used to select the most useful predictors except for the PCAT thickness indexes in the standard model. The optimal λ in the LASSO Cox regression model was determined by 10-fold cross-validation using the one standard error of the minimum criteria (1-SE criteria). Thereafter, the PACT thickness index with the best performance will be added in to update the standard model. The performances of the two models were further assessed by AUCs. The incremental prognostic value of the updated model was evaluated using delta AUC. The category-free net reclassification improvement (NRI) and integrated discrimination improvement (IDI) were calculated as well (16). NRI is defined as the difference in the proportions of study subjects with events correctly assigned a higher probability and study subjects without events correctly assigned a low probability by an updated model compared with the standard one (16). IDI refers to the extent to which the updated model can improve the average sensitivity but without reducing average specificity as compared with that of the standard model (17). Finally, a nomogram that enables the visualization of the probability of 1-year MACE was drawn to assist risk stratification.

A two-tailed *p* < 0.05 was considered statistically significant. All statistical analyses were conducted using R software (version 3.6.3, Vienna, Austria). The following packages were involved: “timeROC,” “survivalROC,” “survival,” “survminer,” “glmnet,” and “survIDINRI.”

## Results

### Baseline Characteristics

Table 1 shows the baseline clinical and CMR imaging characteristics of the study population categorized as control vs. patients with STEMI and further stratified according to the occurrence of MACE. There were no significant differences between the patients with STEMI and the control subjects with respect to age and sex. Patients with STEMI were significantly more likely to have traditional cardiovascular risk factors than the control subjects. Compared with patients without MACE, patients with MACE-STEMI had higher Troponin I value. However, there was no significant difference between patient with and without MACE in peak high-sensitivity C-reactive protein (hs-CRP) as a marker of systemic inflammation. Patients with STEMI, especially those with MACE, had a larger chamber size, lower LVEF, larger infarct size, and less deformation, as evaluated using GRS, GCS, and GLS, than the controls (Table 1).

**Table 1:** Baseline characteristics of study population.

**Variables**	**Controls** **(*N* = 35)**	**All STEMI (*N* = 245)**	***P* value**	**MACE-STEMI (*N* = 47)**	**No MACE-STEMI** **(*N* = 198)**	***P* value**
**Clinical characteristics**						
Age (years)	53.89 ± 9.45	55.61 ± 10.52	0.36	56.87 ± 9.99	55.31 ± 10.65	0.36
Male sex (%)	25 (71.43)	197 (80.41)	0.22	38 (80.85)	159 (80.30)	0.93
BMI (kg/m^2^)	22.01 ± 3.44	25.87 ± 3.29	<0.001	25.75 ± 3.17	25.91 ± 3.33	0.77
BSA (m^2^)	1.71 ± 0.23	1.85 ± 0.18	<0.001	1.84 ± 0.17	1.86 ± 0.18	0.68
Diabetes mellitus (%)	3 (8.57)	62 (25.31)	0.03	9 (19.15)	53 (26.77)	0.28
Hypertension (%)	6 (17.14)	108 (44.08)	<0.01	23 (48.94)	85 (42.93)	0.46
SBP (mm Hg)	119.80 ± 17.28	125.69 ± 21.13	0.12	126.94 ± 19.44	125.40 ± 21.55	0.66
TC (mmol/L)	-	4.88 ± 1.13	-	5.00 ± 1.16	4.85 ± 1.12	0.40
TG (mmol/L)	-	2.00 ± 1.82	-	1.69 ± 1.38	2.08 ± 1.91	0.19
HDL-C (mmol/L)	-	1.02 ± 0.32	-	1.12 ± 0.34	1.00 ± 0.31	0.03
LDL-C (mmol/L)	-	3.01 ± 1.03	-	3.19 ± 0.97	2.97 ± 1.04	0.19
^#^Dyslipidemia (%)	-	185 (75.51)	-	33 (70.21)	152 (76.76)	0.35
eGFR (ml/min/1.73 m^2^)	-	96.33 ± 17.12	-	96.33 ± 16.95	96.33 ± 17.13	1.00
Smoking (%)	7 (20.00)	146 (59.59)	<0.001	30 (63.83)	116 (58.59)	0.51
Heart rate on admission (bpm)	69.63 ± 8.32	78.96 ± 17.72	<0.01	82.34 ± 21.43	78.15 ± 16.69	0.15
Killip class >I (%)	0	35 (14.29)	-	10 (21.28)	25 (12.63)	0.13
Symptom onset to reperfusion time (min)	-	304 (190–535)	-	358 (253–573)	300 (175–529)	0.72
Peak creatine kinase MB (ng/ml)	-	126.00 (79.90–205.35)	-	160.10 (89.30–335.60)	126.00 (79.90–168.30)	0.08
Troponin T (ng/ml)	-	4.08 (1.32–10.0)	-	4.26 (2.13–10.00)	4.08 (1.21–11.34)	0.93
Troponin I (ng/ml)	-	31.60 (9.00–54.83)	-	43.51 (17.65–76.06)	27.35 (8.93–41.54)	<0.01
Peak hs-CPR (mg/dL)	-	5.80 (3.0–10.34)	-	7.00 (3.90–10.80)	5.60 (2.68–10.34)	0.26
Anterior infarction (%)	0	108 (44.08)	-	26 (55.32)	82 (41.41)	0.08
Multivessel disease (%)	0	6 (2.45)	-	1 (2.13)	5 (2.53)	0.87
Initial TIMI flow grade >1 (%)	-	66 (26.94)	-	10 (21.28)	56 (28.28)	0.33
Final TIMI flow grade 3 (%)	-	240 (97.96)	-	45 (95.74)	195 (98.48)	0.23
**Medications**						
Aspirin (%)	6 (17.14)	244 (99.59)	<0.001	47 (100)	197 (99.49)	0.63
Statins (%)	8 (22.86)	241 (98.37)	<0.001	45 (95.74)	196 (98.99)	0.12
ACEI/ARB (%)	7 (20.00)	223 (91.02)	<0.001	43 (91.49)	180 (90.91)	0.90
βblocker (%)	4 (11.43)	224 (91.43)	<0.001	41 (87.23)	183 (92.42)	0.25
**Conventional CMR characteristics**						
LVEF (%)	58.49 ± 6.76	50.15 ± 11.93	<0.001	42.36 ± 12.16	51.99 ± 11.13	<0.001
LVEDVi (ml/m^2^)	69.11 ± 10.72	73.84 ± 14.55	0.07	80.47 ± 16.21	72.27 ± 13.71	<0.001
LVESVi (ml/m^2^)	29.03 ± 7.46	37.61 ± 14.61	0.001	47.48 ± 17.47	35.26 ± 12.81	<0.001
LV mass index (g/m^2^)	52.22 ± 10.58	63.37 ± 11.51	<0.001	65.92 ± 12.93	62.76 ± 11.09	0.09
RVEF (%)	44.18 ± 6.51	37.30 ± 13.51	<0.01	34.00 ± 14.28	38.08 ± 13.24	0.06
RVEDVi (ml/m^2^)	57.95 ± 12.19	57.59 ± 12.80	0.88	56.83 ± 14.89	57.77 ± 12.28	0.65
RVESVi (ml/m^2^)	32.69 ± 8.81	35.85 ± 10.23	0.08	37.28 ± 11.73	35.51 ± 9.85	0.29
Edema size (% of LV mass)	-	37.26 ± 13.52	-	38.24 ± 13.89	36.75 ± 13.65	0.50
Microvascular obstruction (%)	-	88 (35.92)	-	20 (42.55)	68 (34.34)	0.29
Infarct size (% of LV mass)	-	13.73 (8.62–19.01)	-	17.04 (11.53–23.78)	12.97 (7.89–18.05)	<0.01
MSI (% of LV mass)	-	22.16 (8.28–33.15)	-	19.25 (3.22–30.14)	23.67 (10.98–33.58)	0.09
**Strain characteristics**						
GRS (%)	34.09 ± 7.56	23.53 ± 7.98	<0.001	18.96 ± 6.93	24.61 ± 7.85	<0.001
GCS (%)	−20.51 ± 2.86	−15.67 ± 3.49	<0.001	−13.56 ± 3.66	−16.17 ± 3.26	<0.001
GLS (%)	−12.69 ± 2.49	−9.30 ± 2.92	<0.0001	−7.84 ± 2.25	−9.64 ± 2.96	<0.001
**PCAT thickness characteristics**						
RAVGi (mm/m^2^)	4.77 ± 1.15	5.99 ± 1.81	<0.001	6.31 ± 1.78	5.93 ± 1.82	0.19
AIVGi (mm/m^2^)	2.27 ± 0.65	2.81 ± 0.84	<0.001	2.66 ± 0.70	2.85 ± 0.86	0.15
LAVGi (mm/m^2^)	3.54 ± 0.81	4.82 ± 1.44	<0.001	4.67 ± 1.27	4.85 ± 1.47	0.45
SIVGi (mm/m^2^)	3.79 ± 1.11	4.98 ± 1.59	<0.001	5.99 ± 1.55	4.75 ± 1.51	<0.001
IIVGi (mm/m^2^)	2.94 ± 0.99	3.10 ± 0.97	0.35	3.18 ± 1.02	3.08 ± 0.96	0.56

*ACEi, Angiotensin-converting enzyme inhibitors; AIVGi, Anterior interventricular groove PCAT thickness index; ARB, Angiotensin Receptor Blockers; BMI, Body mass index; BSA, Body surface area; CMR, Cardiac Magnetic Resonance; eGFR, Estimated glomerular filtration rate; GCS, Global circumferential strain; GLS, Global longitudinal strain; GRS, Global radial strain; HDL-C, High-density lipoprotein cholesterol; IIVGi, Inferior interventricular groove PCAT thickness index; LAD, Left anterior descending; LAVGi, Left atrioventricular groove PCAT thickness index; LCX, Left circumflex; LDL-C, Low-density lipoprotein cholesterol; LVEDVi, left ventricular end-diastolic volumeindex; LVEF, Left ventricular ejection fraction; LVESVi, left ventricular end-systolic volumeindex; MACE, Major adverse cardiac events; MSI, Myocardial salvage index; PCAT, Pericoronary adipose tissue; RAVGi, Right atrioventricular groove PCAT thickness index; RCA, Right coronary artery; RVEDVi, Right ventricular end-diastolic volume index; RVEF, Right ventricular ejection fraction; RVESVi, Right ventricular end-systolic volume index; SBP, Systolic blood pressure; SIVGi, Superior interventricular groove PCAT thickness index; STEMI, ST elevation myocardial Infarction; TC, Total cholesterol; TG, Triglycerides; TIMI, Thrombolysis in myocardial infarction. Categorical variables are expressed as absolute number (percentage); Symptom onset to reperfusion time, Peak creatine kinase MB, Troponin T, Troponin I, Peak hs-CPR, Infarct size and Myocardial salvage index are expressed as median (IQR); All other continuous variables are expressed as mean ± SD*.

# *Participants were diagnosed with dyslipidemia if they met any of the following criteria27: TC ≥ 6.22 mmol/L, TG ≥ 2.26 mmol/L, LDL-C ≥ 4.14 mmol/L, HDL-C < 1.04 mmol/L, or patients who were taking lipid-regulating medications*.

### Pericoronary Adipose Tissue Thickness Index and Main CMR Parameters

The PCAT thickness indices of patients with STEMI were significantly greater than those of controls at all locations measured, except IIVGi (Table 1). However, only SIVGi showed significant difference between patients with MACE and patients without MACE (Table 1). We found that only SIVGi was associated with LVEF, infarct size, and the three strain indices in all patients with STEMI (Table 2). We also noted a significant increase in SIVGi in patients with lower LVEF, larger infarction size, and less deformation (LVEF≥50%: 4.63 ± 1.47 mm/m^2^, LVEF < 50%:5.34 ± 1.63 mm/m^2^, *p* = 0.001; infarction size ≤ 13.73%:4.60 ± 1.38 mm/m^2^, infarction size > 13.73%:5.37 ± 1.69 mm/m^2^, *p* < 0.001; GRS ≥ 22.77%:4.67 ± 1.57 mm/m^2^, GRS < 22.77%:5.30 ± 1.55 mm/m^2^, *p* < 0.01; GCS ≤ −14.2%:4.71 ± 1.52 mm/m^2^, GCS > −14.2%:5.61 ± 1.57 mm/m^2^, *p* < 0.001; GLS ≤ −8.6%:4.79 ± 1.61 mm/m^2^, GLS > −8.6%:5.26 ± 1.53 mm/m^2^, *p* = 0.02) (Supplementary Table 1).

**Table 2:** The bivariate Pearson's correlations between pericoronary adipose tissue (PCAT) thickness index and left ventricular ejection fraction (LVEF), infarct size, left ventricular end-diastolic volume index (LVEDVi), left ventricular end-systolic volume index (LVESVi), and strains in all patients with ST elevation myocardial Infarction (STEMI).

**Pearson correlations**	**RAVGi**	**AIVGi**	**LAVGi**	**SIVGi**	**IIVGi**
LVEF	0.010	0.064	0.034	−0.299^***^	0.075
Infarct size	0.084	0.041	−0.014	0.299^***^	0.144^*^
LVEDVi	0.029	−0.104	−0.003	0.202^***^	−0.012
LVESVi	0.002	−0.097	−0.034	0.292^***^	−0.066
GRS	0.066	0.079	0.098	−0.238^***^	0.065
GCS	−0.035	−0.011	−0.040	0.307^***^	−0.050
GLS	−0.051	−0.066	−0.054	0.159^*^	−0.047

*AIVGi, Anterior interventricular groove PCAT thickness index; GCS, Global circumferential strain; GLS, Global longitudinal strain; GRS, Global radial strain; IIVGi, Inferior interventricular groove PCAT thickness index; LAVGi, Left atrioventricular groove PCAT thickness index; LVEDVi, left ventricular end-diastolic volume index; LVEF, Left ventricular ejection fraction; LVESVi, left ventricular end-systolic volume index; PCAT, Pericoronary adipose tissue; RAVGi, Right atrioventricular groove PCAT thickness index; SIVGi, Superior interventricular groove PCAT thickness index; STEMI, ST elevation myocardial Infarction*.

* *Indicates P < 0.05*;

*** *indicates P < 0.001*.

### The PCAT Thickness Index With Best Discriminative Ability

**Figure 1:**
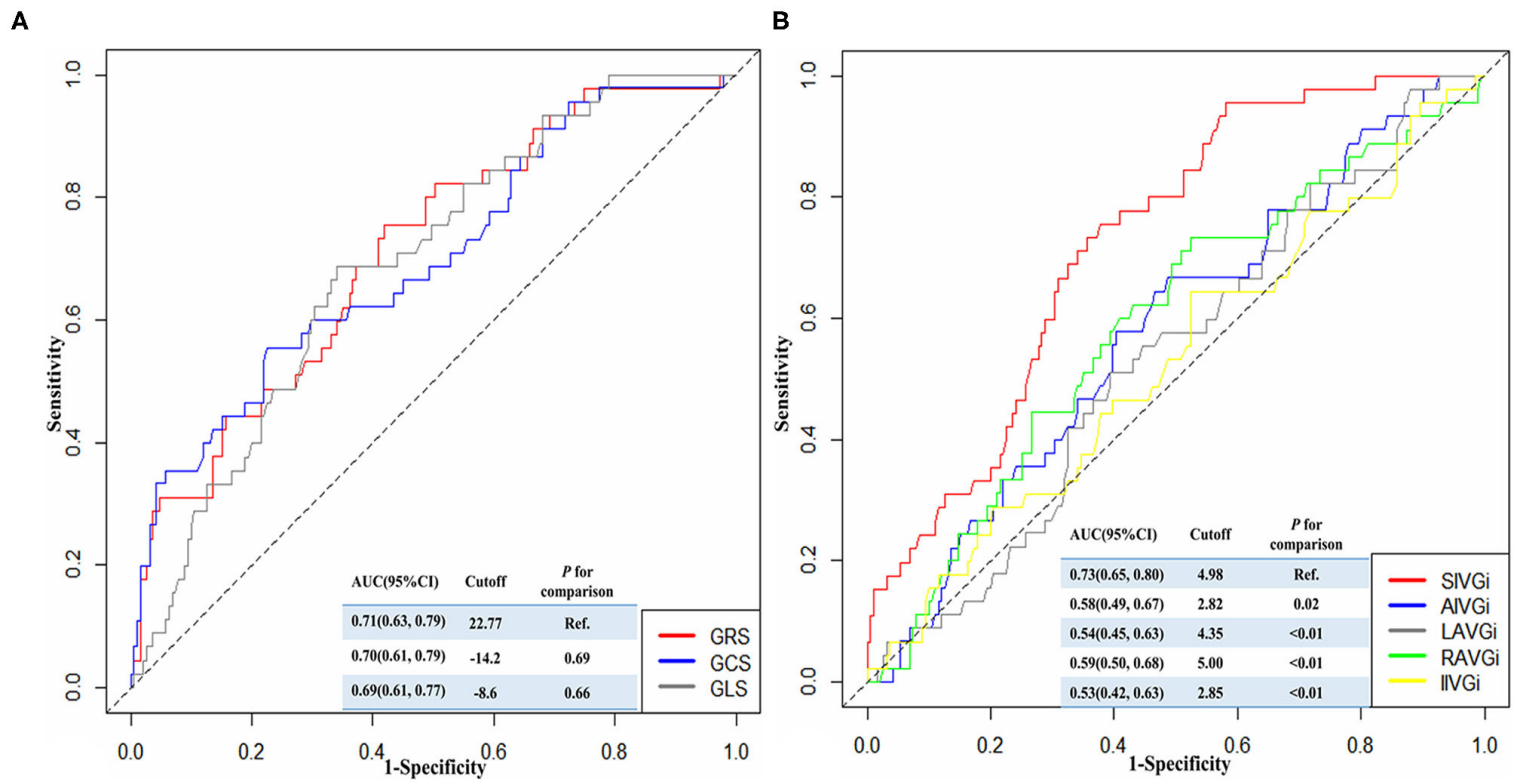
The time-dependent receiver operator characteristic (ROC) curves showing the discriminative ability of different strain characteristics **(A)** and PCAT thickness characteristics **(B)** for predicting 1-year major adverse cardiac events (MACE). AIVGi, Anterior interventricular groove PCAT thickness index; AUC, Areas under the ROC curve; CI, confidence interval; GCS, Global circumferential strain; GLS, Global longitudinal strain; GRS, Global radial strain; IIVGi, Inferior interventricular groove PCAT thickness index; LAVGi, Left atrioventricular groove PCAT thickness index; MACE, Major adverse cardiac events; PCAT, Pericoronary adipose tissue; RAVGi, Right atrioventricular groove PCAT thickness index; ROC, Receiver operator characteristic curve; SIVGi, Superior interventricular groove PCAT thickness index.

**Figure 2:**
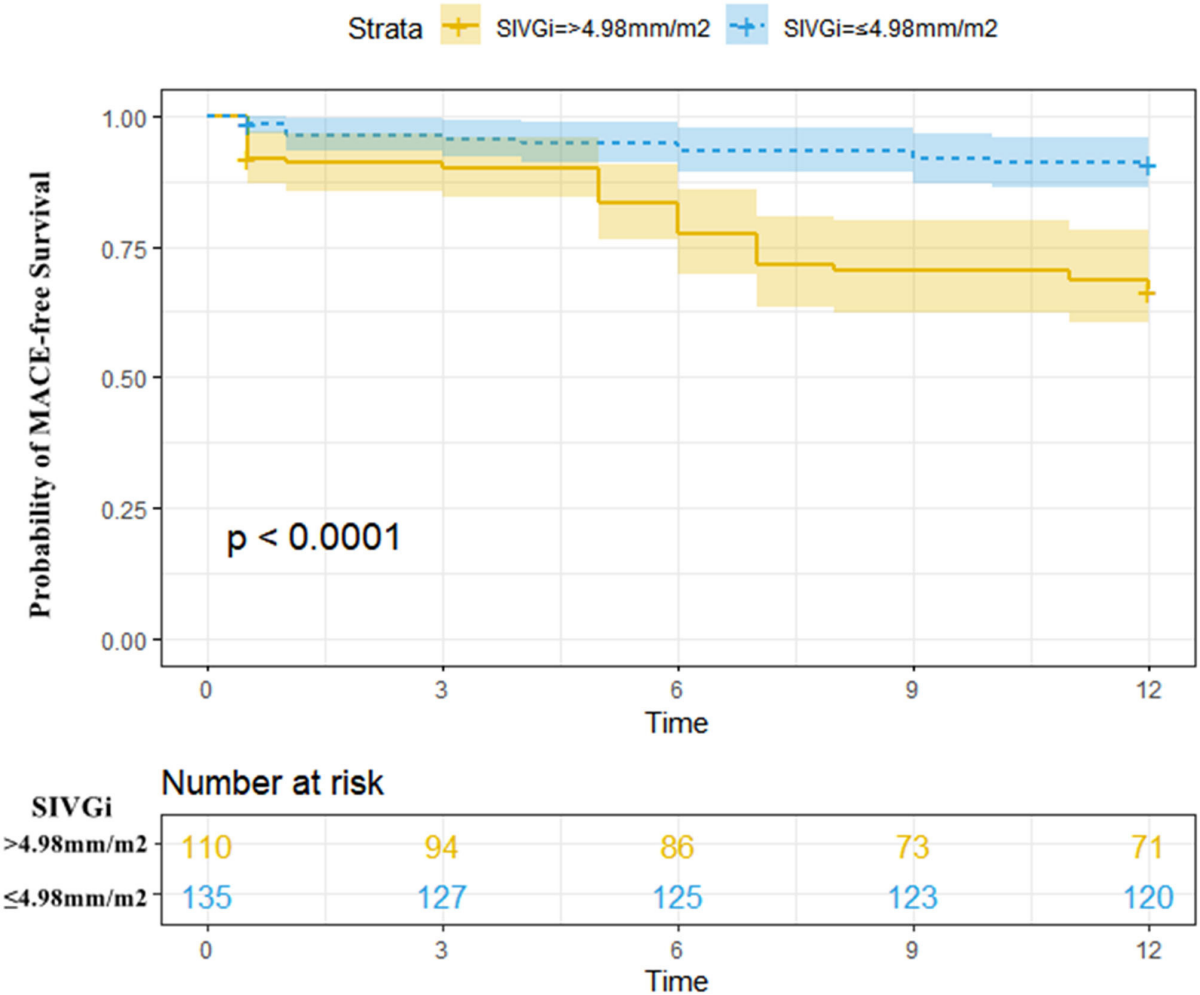
Kaplan–Meier MACE-free survival estimated stratified by SIVGi in all patients with STEMI. Patients with higher SIVGi displayed a significantly higher risk of a first MACE. Central line represented the survival curves and the upper and lower lines of the colored areas correspond to the 95% CI. MACE, Major adverse cardiac events; SIVGi, Superior interventricular groove PCAT thickness index.

During a 1-year follow-up period, 47 patients (19.18%) with STEMI had their first MACE (Supplementary Table 2, Supplementary Figure 5). For all patients, the time-dependent ROC curve analysis demonstrated that the AUC of 0.73 (95% CI: 0.66–0.80, *p* < 0.01, cutoff value: 4.98 mm/m^2^) for SIVGi was the highest among the AUCs of the PCAT thickness indices (Figure 1B). Moreover, the Kaplan–Meier event-free survival curve analysis also showed that SIVGi > 4.98 mm/m^2^ was associated with poor 1-year MACE-free survival in patients with STEMI (Figure 2). Therefore, SIVGi was significant associated with the time to MACE. To avoid variable overfitting, SIVGi was the only PCAT thickness parameter tested in subsequent multivariable analyses.

Similarly, the discriminative ability of different strain characteristics for predicting 1-year MACE was also identified by the time-dependent ROC curve. It was shown that the AUC for GRS was higher among the three parameters, and there was no significant difference compared with the other two AUCs (Figure 1A).

### Univariate and Multivariable Analysis

Univariate Cox regression analysis was performed to estimate the risk predictors of one-year MACE post-STEMI (Supplementary Table 3). It demonstrated that a Troponin *I* > 31.60 ng/ml, a LVEF < 50%, and an infarct size >13.73% were associated with a two- to three-fold increased risk for adverse events, and that a LVESV index > 35.99% ml/m^2^, a GRS < 22.77%, a GCS > −14.2%, and a GLS > −8.6% were associated with an approximately four-fold increased risk for adverse events for all the patients with STEMI (Supplementary Table 3). Further, SIVGi > 4.98 mm/m^2^ was associated with an approximately five-fold increased risk for MACE (Supplementary Table 3).

The standard model in Table 3 showed that LVESV index, GRS, GCS, and GLS were significantly associated with 1.89-, 1.77-, 1.86-, and 2.03-fold elevated risk of adverse events, respectively (Supplementary Figure 3). The addition of SIVGi in the updated model revealed that SIVGi was independently associated with risk of adverse events (*HR* = 3.2, 95% *CI*: 1.6–6.38, *p* < 0.001). Similar results were found when LVESV index, GRS, GCS, GLS, and SIVGi were enrolled into Cox regression model as continuous variables. There was a 1.49-fold higher risk in adverse events per mm/m^2^ increase in SIVGi (Supplementary Table 4).

**Table 3:** Comparison of the standard and updated multivariate Cox regression models for predicting 1-year major adverse cardiac events (MACE) post STEMI.

**Variables**	**Standard model**		**Updated model**	
	**HR (95%CI)**	***P* value**	**HR (95%CI)**	***P* value**
LVESVi (>35.99 ml/m^2^)	1.89 (0.89, 4.00)	0.10	1.87 (0.90, 3.87)	0.09
GRS (<22.77%)	1.77 (0.80, 3.94)	0.16	1.67 (0.75, 3.68)	0.21
GCS (>-14.2%)	1.86 (0.93, 3.71)	0.08	1.53 (0.77, 3.02)	0.23
GLS (>-8.6%)	2.03 (1.03, 4.01)	0.04	1.98 (1.00, 3.90)	0.05
SIVGi (>4.98 mm/m^2^)	-	-	3.20 (1.60, 6.38)	<0.001
AUC (95%CI)	0.76 (0.68, 0.83)		0.80 (0.74, 0.87)	
Delta AUC; *P* value	0.046; 0.047			
cfNRI (95%CI); *P* value	0.38 (0.10, 0.53); 0.01			
IDI (95%CI); *P* value	0.09 (0.01, 0.18); 0.02			

*AUC, Areas under the ROC curve; cfNRI, category-free net reclassification index; CI, Confidence interval; GCS, Global circumferential strain; GLS, Global longitudinal strain; GRS, Global radial strain.HR, Hazard ratio; IDI, Integrated discrimination improvement index; LVEF, Left ventricular ejection fraction; LVESVi, left ventricularend-systolic volume index; MACE, Major adverse cardiac events; SIVGi, Superior interventricular groove; PCAT thickness index; STEMI, ST elevation myocardial Infarction*.

### Incremental Discriminatory and Reclassification Performance of SIVGi

As shown in Table 3, the discriminative power of the updated model was stronger than that of the standard model (*AUC*: 0.8 *vs*. 0.76). The delta *AUC* and *p*-value were 0.046 and 0.047, respectively. The incremental prognostic value of SIVGi was further confirmed by reclassification analysis. As compared with the standard model, the updated model could more accurately assigned 38% of the participants into the MACE group or non-MACE group (category-free NRI [*cfNRI*] = 0.38, *p* = 0.01), which was consistent with the non-zero IDI value (*IDI* = 0.09, *p* = 0.02). Similar findings were depicted in the sensitivity analysis in which traditional risk factors such as age, sex, smoking, BMI, diabetes mellitus, hypertension, dyslipidemia, eGFR, LVEF, statins, ACEI/ARB, and β blocker were further adjusted (Supplementary Table 5).

A higher AUC was also found in the updated model than the standard model when the SIVGi was treated as a continuous variable in Cox regression models (*AUC*: 0.82 vs. 0.74; delta *AUC* = 0.08, *p* < 0.01) (Supplementary Table 4). As compared with the standard model, the updated model could more accurately assigned 29% of the participants (*cfNRI* = 0.29, *p* = 0.03). However, we failed to detect a significant IDI value (*IDI* = 0.03, *p* = 0.31).

Based on the updated model, a nomogram that combined all the independent predictors was constructed to predict MACE at 1 year (Supplementary Figure 4). In this nomogram, each variable was given a score, and by summing up the total score of each patient, we could predict the possibility of MACE at 1 year.

## Discussion

The main finding of the present study was that the increased PCAT thickness at the SIVG was more significant in patients with MACE after STEMI. The SIVGi was significantly associated with LVEF, infarct size and LV global deformation in STEMI. As an independent predictor for 1-year MACE, SIVGi conferred incremental value in predictions for patients with STEMI.

Pericoronary adipose tissue as an organ is anatomically adjacent and functionally interrelated with the myocardium and coronary arteries. Under physiological conditions, PCAT has anti-inflammatory and protective effects and can be used as an energy reservoir to provide fatty acids to the coronary arteries and myocardium (18–20). However, PCAT contains a high density of lymphoid clusters and as a chemical reservoir, can secrete pro-inflammatory cytokines and directly induce adjacent coronary artery inflammation in pathological states, thereby promoting the progression of atherosclerosis, plaque rupture, and thrombosis, causing acute MI (9, 21, 22). Acute MI can induce the pathological state of EAT. It is well known that as a cardiovascular disease that increases sympathetic activation, MI is characterized by sustained stimulation of β-adrenoceptors (β-ARs) and activation of the renin-angiotensin-aldosterone system. The vascular dysfunction induced by the overstimulation of β-AR associated with endothelial nitric oxide synthase uncoupling can cause PCAT impairment (23). At the same time, the infarcted myocardium directly secretes inflammatory factors and causes hypoxia in the surrounding microenvironment to promote the inflammatory response of the adjacent EAT, which can promote accumulation and thickening of local EAT (24). The findings of the present study are in accordance with the global and local effects of MI on EAT. We found that for patients with STEMI, PCAT thickened in all locations, and local PCTA at SIVG more significantly thickened in patient with MACE. However, in our study, the peak hs-CPR, as a marker of systemic inflammation, was not significantly increase in patient with MACE compared with patients without MACE. It supported that the local PCAT could be a more direct and efficient indicator on MACE after STEMI compared with global changes.

Previous ultrasound studies have explored the relationship between local EAT thickness and the prognosis of patients with MI (25, 26). Morales-Portano et al. used ultrasound to measure the mean EAT thickness at the right ventricle free wall and the interventricular groove, which showed the good predictive ability for MACE in patients with CAD (25). However, in a 3-year follow-up study of 114 patients with STEMI, Esen et al. found that the EAT thickness at the right ventricle free wall was not related to the clinical outcome after PCI (26). Therefore, the prognostic value of local EAT thickness for STEMI remains controversial. In the present study, we found that SIVGi is a stronger predictor of outcome than other PCAT thickness parameters. An SIVGi > 4.98 mm/m^2^ was associated with a 4.52-fold increased risk for MACE after STEMI, whereas other PCAT thickness indices were associated with about 1- to 2-fold increased risk for MACE. A previous CT study indicated that PCAT around the left anterior descending (LAD) artery and right coronary artery, but not around the left circumflex coronary artery, is an independent predictor of all-cause and cardiac mortality (11), a finding that supports that of the present study. This is also consistent with the result of another recent MR study, which showed that perivascular EAT at the SIVG is an independent predictor of composite MACE in patients with STEMI (odds ratio, 2.26) after adjustment for age, sex, and LVEF (13).

The findings of the present study and those of previous studies indicate that there are some possible reasons for the significant thickening of PCAT at the SIVG and why it is an independent predictor of MACE. First, the interventricular groove, which is shallow and broad, is where the EAT has the closest contact and is most adjacent to the myocardium, especially the EAT at the SIVG, which is most directly affected by paracrine effects produced by the anterior infarcted myocardium. This could be supported by the significant increase in SIVGi in MACE group, as there were more patients with anterior MI (~60%) and larger MI sizes in MACE-STEMI compared with no MACE-STEMI in the present study. Second, given that LAD is the main artery of the left ventricle with the most extensive blood supply range, the redistribution of coronary blood flow is most significant after MI. In addition, the damage to the endothelial cells of the LAD can induce more obvious abnormal accumulation of PCAT around the LAD. The effect of PCAT at the SIVG on blood vessels is also stronger and has greater influence on prognosis (27). Third, according to the characteristics of the anatomical location, the measurement value of PCAT thickness at the SIVG is more accurate, more reproducible, and more sensitive to the pathological state of the PCTA than that of other locations. Compared with other PCAT thickness indices, SIVGi had perfect repeatability and the highest AUC value in the present study, a finding that further supports this view. Therefore, it is not appropriate to analyze the overall change in EAT as doing so may dilute the changes controlled by PCAT at the SIVG, which may reduce the ability of PCAT to assess prognosis. Indeed, we found that the higher SIVGi was significantly assistant with lower LVEF, larger LV infarct area, and less deformation, all of which indicate a poor prognosis in patients with STEMI. The deterioration of these parameters may suggest the pathologic effect of PCAT in patients with STEMI.

The results of the present study showed that compared with standard model, the updated model including SIVGi with a cutoff value of 4.98 mm/m^2^ had better discrimination and reclassification ability for patients with 1-year MACE after STEMI. The sensitivity analysis in which traditional risk factors were further adjusted also supported this. Interestingly, the most useful predictors (GRS, GCS, GLS, and LVESV) for the standard model are all CMR parameters for myocardial compliance, indicating that myocardial compliance on CMR imaging may have more direct relationship with short-term STEMI prognosis compared with clinical parameters. To our knowledge, this is the first study to show that the simple measurement of PCAT thickness index at the SIVG combined with myocardial compliance indices is a useful marker of MACE after STEMI in the CMR imaging setting. Moreover, PCAT measurement has good reliability and can be completed during routine CMR examination without the need for a special sequence and complex post-processing software. Therefore, the PCAT thickness index is an ideal prognostic indicator.

In this study, the MACE was observed in 19.18% of patients. The similar result is also appeared in recent study from Zhang et al. (28), 1-year follow-up MACE in STEMI patients was 20.4%. However, the incidence of MACE was slightly higher than others. The limited number of cases included in our study and regional differences may be one of the main reasons (29). The wide definition of MACE also increased the incidence, as in this study, patients with heart failure hospitalization were included, accounting for 42%. In addition, more than 75% of patients in this study had dyslipidemia, and 83.27% of patients had pain-to-balloon time more than 120 min, which could lead to a poor prognosis (30, 31).

This study has several limitations. First, the retrospective and observational cohort design of the study makes it challenging to derive causality. Second, PCAT inflammation can be caused by percutaneous endothelium injury during endovascular angioplasty (32). It is difficult to estimate the effect of this procedure on the inflammation of PCAT. However, all the PCAT measurements were taken at different locations; the observed effect of PCAT on MACE could not be explained by the procedure-associated inflammation of PCAT alone. Third, only PCAT thickness was measured in this study. Therefore, the relationship between PCAT thickness and EAT volume needs to be studied further. However, a previous study by Alam et al. showed that the thickness of EAT can reflect myocardial microvascular dysfunction more than the volume of EAT (33). Finally, we used the multivariate analysis method to calculate the 1-year MACE prediction model including PCAT. As the model was obtained from a small sample, its validity needs to be verified further.

## Conclusion

Patients with MACE have more significant increase in PCAT thickness at SIVG than without MACE after STEMI. PCAT thickness index at the SIVG could possibly be a strong independent predictor of MACE in revascularized patients with STEMI. Compared with standard model based on clinical and CMR parameters, the updated model including SIVGi has significantly improved predictive power for 1-year MACE after STEMI.

## Data Availability Statement

The original contributions presented in the study are included in the article/Supplementary Material, further inquiries can be directed to the corresponding author.

## Ethics Statement

The studies involving human participants were reviewed and approved by Shengjing Hospital of China Medical University Research Ethics Committee. Written informed consent for participation was not required for this study in accordance with the national legislation and the institutional requirements.

## Author Contributions

YM, YH, and QM participated in the study design. XW, TY, YD, and JS participated in data collection. GL performed the statistical analysis. YM drafted the article. All authors contributed to the article and approved the submitted version.

## Funding

This study has received funding by the National Natural Science Foundation of China (Grant Nos. 81901741 and 82071920), the Key Research and Development Plan of Liaoning Province (No. 2020JH2/10300037), and the Talent Project in the Shengjing Hospital of China Medical University.

## Conflict of Interest

The authors declare that the research was conducted in the absence of any commercial or financial relationships that could be construed as a potential conflict of interest.

## Publisher's Note

All claims expressed in this article are solely those of the authors and do not necessarily represent those of their affiliated organizations, or those of the publisher, the editors and the reviewers. Any product that may be evaluated in this article, or claim that may be made by its manufacturer, is not guaranteed or endorsed by the publisher.
