# Supplementary material for: Incremental Prognostic Value of Pericoronary Adipose Tissue Thickness Measured Using Cardiac Magnetic Resonance Imaging After Revascularization in Patients With ST-Elevation Myocardial Infarction
Source: Front Cardiovasc Med. 2022 Mar 4;9:781402. doi: 10.3389/fcvm.2022.781402 (PMC8934413; doi:10.3389/fcvm.2022.781402)
Supplement: Supplementary file 1 [file Data_Sheet_1.docx]

Supplementary Material

**Supplemental Table 1.** Comparisons of PCAT thickness indices characteristics in all STEMI patients with different LVEF, infarction size and strains level.

| Variables  （mm/m^2^） | LVEF | | *P*  Value | Infarct size | | *P* Value | GRS | | *P*  Value | GCS | | *P*  Value | GLS | | *P*  Value |
| --- | --- | --- | --- | --- | --- | --- | --- | --- | --- | --- | --- | --- | --- | --- | --- |
|  | ≥50% | <50% |  | ≤13.73% | >13.73% |  | ≥22.77% | <22.77% |  | ≤ -14.2% | >-14.2% |  | ≤-8.6% | >-8.6% |  |
| RAVGi | 5.99±1.68 | 6.00±1.94 | 0.96 | 5.87±1.70 | 6.13±1.91 | 0.26 | 6.10±1.68 | 5.90±1.94 | 0.43 | 6.03±1.75 | 5.92±1.96 | 0.64 | 6.04±1.79 | 5.95±1.85 | 0.70 |
| AIVGi | 2.86±0.86 | 2.77±0.81 | 0.42 | 2.73±0.87 | 2.89±0.80 | 0.13 | 2.87±0.86 | 2.75±0.81 | 0.29 | 2.85±0.84 | 2.85±0.84 | 0.29 | 2.83±0.90 | 2.79±0.74 | 0.72 |
| LAVGi | 4.85±1.47 | 4.79±1.41 | 0.72 | 4.80±1.41 | 4.84±1.46 | 0.82 | 4.97±1.52 | 4.66±1.34 | 0.08 | 4.80±1.45 | 4.86±1.42 | 0.79 | 4.76±1.51 | 4.90±1.33 | 0.48 |
| **SIVGi** | **4.63±1.47** | **5.34±1.63** | **0.001** | **4.60±1.38** | **5.37±1.69** | **<0.001** | **4.67±1.57** | **5.30±1.55** | **<0.01** | **4.71±1.52** | **5.61±1.57** | **<0.001** | **4.79±1.61** | **5.26±1.53** | **0.02** |
| IIVGi | 3.17±1.05 | 3.04±0.89 | 0.30 | 2.97±0.94 | **3.23±0.99** | **0.03** | 3.16±1.07 | 3.04±0.87 | 0.40 | 3.13±1.02 | 3.04±0.85 | 0.56 | 3.16±1.01 | 3.02±0.92 | 0.30 |

**Abbreviations**. AIVGi= Anterior interventricular groove PCAT thickness index.GCS=Global circumferential strain. GLS=Global longitudinal strain.GRS=Global radial strain. IIVGi= Inferiorinterventricular groove PCATthickness index.LAVGi=Left atrioventricular groove PCAT thickness index. LVEDVi= left ventricular end-diastolic volumeindex. LVEF=Left ventricular ejection fraction. LVESVi= left ventricular end-systolic volumeindex. PCAT=Pericoronaryadipose tissue. RAVGi=Rightatrioventricular groove PCAT thickness index.SIVGi= Superior interventricular groove PCAT thickness index.STEMI=STelevation myocardial Infarction.

| **Supplemental Table 2.** Individual MACE during follow-up. | MACE(n=47)/ All-STEMI (n=245) |
| --- | --- |
| Cardiovascular death, N (%) | 5 (10.64) |
| Recurrent myocardial infarction, N (%) | 17 (36.17) |
| Target lesion revascularization, N (%) | 5 (10.64) |
| Heart failure hospitalization, N (%) | 20 (42.55) |

**Abbreviations**.STEMI=STelevation myocardial Infarction. MACE=Major adverse cardiac events.

**Supplemental Table 3.** The exploration of predictors for one-year MACE among STEMI patients using univariate Cox regression analysis.

| **Variables** | **HR** | **95% CI** | ***P* value** |
| --- | --- | --- | --- |
| **Clinical characteristics** |  |  |  |
| Age (≥60years) | 1.34 | 0.76-2.37 | 0.32 |
| Male *vs.* female | 1.05 | 0.51-2.18 | 0.89 |
| BMI (≥24.0kg/m^2^) | 1.07 | 0.55-2.05 | 0.85 |
| BSA (>1.88m^2^) | 0.85 | 0.48-1.52 | 0.59 |
| Diabetes mellitus | 0.67 | 0.33-1.39 | 0.29 |
| Hypertension | 1.22 | 0.69-2.17 | 0.49 |
| Dyslipidemia^#^ | 0.72 | 0.39-1.35 | 0.31 |
| eGFR<60 ml/min/1.73m^2^ | 0.98 | 0.53-1.81 | 0.95 |
| Smoking | 1.29 | 0.71-2.35 | 0.40 |
| Heart rate on admission (>80bpm) | 1.12 | 0.62-2.01 | 0.71 |
| Killip class >I | 2.01 | 1.00-4.04 | 0.05 |
| Symptom onset to reperfusion time (>360min) | 1.27 | 0.72-2.25 | 0.42 |
| Peak creatine kinase MB (>126ng/ml) | 1.77 | 0.99-3.17 | 0.06 |
| Troponin T (>4.08ng/ml) | 1.07 | 0.61-1.90 | 0.81 |
| Troponin I (>31.60ng/ml) | 2.35 | 1.31-4.24 | <0.01 |
| Peak hs-CPR (>5.80mg/dL) | 1.40 | 0.79-2.48 | 0.26 |
| Anterior infarction (yes *vs.* no) | 1.72 | 0.97-3.05 | 0.07 |
| Multivessel disease (yes *vs.* no) | 0.88 | 0.12-6.40 | 0.90 |
| Initial TIMI flow grade >1 | 1.39 | 0.69-2.80 | 0.35 |
| Final TIMI flow grade 3 | 2.34 | 0.57-9.64 | 0.24 |
| **Medications** |  |  |  |
| Statins (use) | 0.28 | 0.07-1.17 | 0.08 |
| ACEI/ARB (use) | 1.05 | 0.38-2.92 | 0.93 |
| *β* blocker (use) | 0.56 | 0.24-1.34 | 0.20 |
| **Conventional CMR characteristics** |  |  |  |
| LVEF (<50.0%) | 2.67 | 1.43-4.99 | <0.01 |
| LVEDVi (>73.32ml/m²) | 1.98 | 1.09-3.60 | 0.02 |
| LVESVi (>35.99ml/m²) | 3.56 | 1.81-6.99 | <0.001 |
| LV mass index (>62.48g/m^2^) | 1.40 | 0.78-2.49 | 0.26 |
| RVEF (<38.35%) | 1.75 | 0.97-3.14 | 0.06 |
| RVEDVi (<57.86ml/m²) | 1.42 | 0.80-2.54 | 0.23 |
| RVESVi (>35.73ml/m²) | 1.27 | 0.71-2.25 | 0.42 |
| Edema size (>36.26% of LV mass) | 1.38 | 0.78-2.47 | 0.27 |
| Microvascular obstruction (yes *vs.* no) | 1.36 | 0.76-2.43 | 0.30 |
| Infarct size (>13.73% of LV mass) | 2.40 | 1.30-4.43 | <0.01 |
| MSI (>22.77% of LV mass) | 1.72 | 0.96-3.10 | 0.07 |
| **Strain characteristics** |  |  |  |
| GRS (<22.77%) | 3.89 | 1.98-7.64 | <0.001 |
| GCS (>-14.2%) | 3.96 | 2.22-7.06 | <0.001 |
| GLS (>-8.6%) | 3.60 | 1.95-6.65 | <0.001 |
| **PCAT thickness characteristics** |  |  |  |
| RAVGi (>5.00 mm/m^2^) | 1.53 | 0.81-2.90 | 0.19 |
| AIVGi (<2.82mm/m^2^) | 1.87 | 1.02-3.42 | 0.04 |
| LAVGi (>4.35mm/m^2^) | 0.66 | 0.37-1.17 | 0.15 |
| SIVGi (>4.98 mm/m^2^) | 4.52 | 2.30-8.89 | <0.001 |
| IIVGi (>2.85mm/m^2^) | 1.50 | 0.83-2.72 | 0.18 |

**^#^** Participants were diagnosed with dyslipidemia if they met any of the following criteria: TC≥6.22 mmol/L, TG≥2.26 mmol/L, LDL-C≥4.14 mmol/L, HDL-C<1.04 mmol/L, or patients who were taking lipid-regulating medications.

**Abbreviations**. ACEi=Angiotensin-converting enzyme inhibitors.AIVGi=Anterior interventricular groove PCAT thickness index. ARB=Angiotensin Receptor Blockers. BMI=Body mass index. BSA=Body surface area. CMR=Cardiac Magnetic Resonance. eGFR= Estimated glomerular filtration rate. GCS=Global circumferential strain. GLS=Global longitudinal strain. GRS=Global radial strain. IIVGi = Inferior interventricular groove PCAT thickness index. LAD=Left anterior descending. LAVGi= Left atrioventricular groove PCAT thickness index. LCX=Left circumflex.LVEDVi= left ventricular end-diastolic volumeindex. LVEF=Left ventricular ejection fraction. LVESVi= left ventricular end-systolic volumeindex. MACE=Major adverse cardiac events. MSI=Myocardial salvage index. PCAT= Pericoronary adipose tissue. RAVGi= Right atrioventricular groove PCAT thickness index. RCA=Right coronary artery. RVEDVi= Right ventricular end-diastolic volume index. RVEF= Right ventricular ejection fraction. RVESVi= Right ventricular end-systolic volume index. SIVGi = Superior interventricular groove PCAT thickness index.STEMI=ST elevation myocardial Infarction.TIMI=Thrombolysis in myocardial infarction. HR=hazard ratios. CI=confidence interval.

**Supplemental Table 4.** Comparison of the standard and updated multivariate Cox regression models^#^ for predicting 1-year MACE post STEMI.

| **Variables** | **Standard model** | |  | **Updated model** | |
| --- | --- | --- | --- | --- | --- |
|  | **HR (95%CI)** | ***P* value** |  | **HR (95%CI)** | ***P* value** |
| **LVESVi (**ml/m²**)** | 1.03 (1.00, 1.05) | 0.03 |  | 1.02 (1.00, 1.05) | 0.06 |
| **GRS (%)** | 0.97 (0.90, 1.03) | 0.30 |  | 0.96 (0.89,1.02) | 0.17 |
| **GCS (%)** | 1.04 (0.88, 1.23) | 0.61 |  | 0.97 (0.82,1.14) | 0.30 |
| **GLS (%)** | 1.05 (0.92, 1.21) | 0.45 |  | 1.08 (0.94, 1.24) | 0.08 |
| **SIVGi (mm/m^2^)** | - | - |  | 1.49 (1.23,1.81) | <0.001 |
| **AUC (95%CI)** | 0.74 (0.67, 0.82) | |  | 0.82 (0.76, 0.88) | |
| **Delta AUC; *P* value** | 0.08; <0.01 | | | | |
| **cfNRI (95%CI); *P* value** | 0.29 (0.01, 0.42); 0.03 | | | | |
| **IDI (95%CI); *P* value** | 0.03 (-0.03, 0.10); 0.31 | | | | |

^#^ LVESVi, GRS, GCS, GLS, and SIVGi as continuous variables.

**Abbreviations:** AUC=Areas under the ROC curve. cfNRI=Category-free net reclassification index. CI=Confidence interval. GCS=Global circumferential strain. GLS=Global longitudinal strain. GRS=Global radial strain.HR=Hazard ratio. IDI=Integrated discrimination improvement index. LVESVi=left ventricularend-systolic volume index. MACE=Major adverse cardiac events. SIVGi= Superior interventricular groove PCAT thickness index.STEMI=STelevation myocardial Infarction.

**Supplemental Table 5.** Comparison of the standard and updated multivariate Cox regression models for predicting 1-year MACE post STEMI.

| **Variables** | **Standard model** | |  | **Updated model** | |
| --- | --- | --- | --- | --- | --- |
|  | **HR (95%CI)** | ***P* value** |  | **HR (95%CI)** | ***P* value** |
| **LVESVi (>35.99ml/m²)** | 2.45 (0.99, 6.03) | 0.05 |  | 2.23 (0.94, 5.3) | 0.07 |
| **GRS (<22.77%)** | 1.95 (0.85, 4.48) | 0.12 |  | 1.86 (0.81, 4.24) | 0.14 |
| **GCS (>-14.2%)** | 2.14 (0.98, 4.66) | 0.06 |  | 1.78 (0.80, 3.95) | 0.16 |
| **GLS (>-8.6%)** | 2.29 (1.13, 4.66) | 0.02 |  | 2.29 (1.12, 4.68) | 0.02 |
| **Age (≥60years)** | 1.41 (0.72, 2.74) | 0.32 |  | 1.05 (0.54, 2.05) | 0.89 |
| **Male *vs.* female** | 1.24 (0.53, 2.93) | 0.62 |  | 1.12 (0.46, 2.7) | 0.80 |
| **BMI (≥24.0kg/m^2^)** | 1.36 (0.66, 2.82) | 0.41 |  | 1.66 (0.78, 3.52) | 0.19 |
| **Diabetes mellitus** | 0.49 (0.22, 1.10) | 0.08 |  | 0.52 (0.22, 1.21) | 0.13 |
| **Hypertension** | 1.90 (1.00, 3.59) | 0.05 |  | 1.78 (0.93, 3.40) | 0.08 |
| **Dyslipidemia**^#^ | 0.72 (0.36, 1.45) | 0.36 |  | 0.74 (0.37, 1.48) | 0.39 |
| **eGFR<60 ml/min/1.73m^2^** | 2.98(0.31, 28.48) | 0.34 |  | 2.90 (0.33, 25.85) | 0.34 |
| **Smoking** | 1.26 (0.64, 2.46) | 0.50 |  | 1.52 (0.77, 3.02) | 0.23 |
| **Statins (use)** | 0.21 (0.03, 1.34) | 0.10 |  | 0.26 (0.04, 1.69) | 0.16 |
| **ACEI/ARB (use)** | 1.57 (0.44, 5.56) | 0.49 |  | 1.49 (0.38, 5.75) | 0.57 |
| ***β* blocker (use)** | 0.72 (0.25, 2.10) | 0.55 |  | 0.76 (0.25, 2.32) | 0.63 |
| **LEVF (<50%)** | 0.62 (0.25, 1.50) | 0.29 |  | 0.60 (0.25, 1.47) | 0.26 |
| **SIVGi (>4.98 mm/m^2^)** | - | - |  | 3.28 (1.57, 6.84) | <0.01 |
| **AUC (95%CI)** | 0.78 (0.71, 0.86) | |  | 0.82 (0.76, 0.89) | |
| **Delta AUC; *P* value** | 0.041; 0.02 | | | | |
| **cfNRI (95%CI); *P* value** | 0.39 (0.06, 0.57); 0.02 | | | | |
| **IDI (95%CI); *P* value** | 0.08 (0, 0.17); 0.03 | | | | |

^#^ Participants were diagnosed with dyslipidemia if they met any of the following criteria: TC≥6.22 mmol/L, TG≥2.26 mmol/L, LDL-C≥4.14 mmol/L, HDL-C<1.04 mmol/L, or patients who were taking lipid-regulating medications.

**Abbreviations:** AUC=Areas under the ROC curve. cfNRI=Category-free net reclassification index. CI=Confidence interval. eGFR= Estimated glomerular filtration rate. GCS=Global circumferential strain. GLS=Global longitudinal strain. GRS=Global radial strain.HR=Hazard ratio. IDI=Integrated discrimination improvement index. LVEF=Left ventricular ejection fraction. LVESVi=left ventricularend-systolic volume index. MACE=Major adverse cardiac events. SIVGi= Superior interventricular groove PCAT thickness index. STEMI=STelevation myocardial Infarction.

**Reproducibility**

The intra- and inter-observer variabilities of the CMR imaging parameters and PCAT indices were assessed in 15 subjects randomly selected from the patient and control groups. For intra-observer variability, the anonymized data sets were analyzed again by a blinded observer (Q.M.) one month after the initial measurements were taken. Inter-observer variability was determined by analyzing anonymized data from two separate observers (Q.M. and Y.M.) blinded to each other’s results.

Supplemental Table 7 shows the intra- and inter-observer variabilities. All CMR imaging parameters, including PCAT thickness indices, had intra- and inter-observer intraclass correlation coefficients of ≥ 0.85. SIVGi demonstrated an intra-observer bias of -0.231 (95% limits of agreement, -0.174 to 0.635) and an inter-observer bias of -0.641 (95% limits of agreement, -0.893 to -0.388).

**Supplemental Table 6A.** Inter-observer variability for CMR indices and PCAT indices.

| **Variable** | **Bland-Altman analysis** | | **Coefficient of variation** | **Intra-class correlation coefficient** |
| --- | --- | --- | --- | --- |
|  | **Bias** | **95% limits of agreement** |  |  |
| **LVEF (%)** | 0.716 | -0.584- 2.016 | 0.051 | 0.980 |
| **LV end-diastolic volume index (ml/m²)** | 0.438 | -2.531- 3.407 | 0.065 | 0.934 |
| **LV end-systolic volume index (ml/m²)** | -2.404 | -4.013 - -0.796 | 0.061 | 0.973 |
| **LV mass (g/m^2^)** | -1.445 | -3.406 - 0.516 | 0.050 | 0.886 |
| **RVEF (%)** | 0.121 | -1.456 - 1.697 | 0.075 | 0.986 |
| **RV end-diastolic volume index (ml/m²)** | -0.118 | -2.145- 1.908 | 0.063 | 0.977 |
| **RV end-systolic volume index (ml/m²)** | -0.425 | -0.611 - -0.239 | 0.050 | 0.992 |
| **Infarct size (% of LV mass)** | 0.048 | -0.621- 0.716 | 0.072 | 0.991 |
| **Edema (% of LV mass)** | -0.700 | -2.184 - 0.784 | 0.062 | 0.964 |
| **Myocardial salvage index (% of LV mass)** | -0.265 | -1.194 - 0.665 | 0.078 | 0.993 |
| **GRS (%)** | -0.357 | -0.953- 0.239 | 0.058 | 0.973 |
| **GCS (%)** | -0.545 | -0.915 - -0.174 | 0.048 | 0.968 |
| **GLS (%)** | -0.484 | -0.649- -0.318 | 0.037 | 0.971 |
| **RAVGi (mm/m^2^)** | -0.425 | -0.611 - -0.239 | 0.048 | 0.915 |
| **AIVGi (mm/m^2^)** | -0.459 | -0.608 - -0.310 | 0.087 | 0.855 |
| **LAVGi (mm/m^2^)** | -0.513 | -0.666 -0.360 | 0.056 | 0.871 |
| **SIVGi (mm/m^2^)** | -0.641 | -0.893 - -0.388 | 0.064 | 0.942 |
| **IIVGi (mm/m^2^)** | -0.479 | -0.631 - -0.327 | 0.066 | 0.891 |

**Abbreviations.** PCAT=Pericoronaryepicardial adipose tissue. STEMI= STelevation myocardial Infarction. MACE=Major adverse cardiac events.LVEF=Left ventricular ejection fraction. RVEF=Right ventricular ejection fraction. RAVGi=Rightatrioventricular groovePCAT index. LAVGi=Left atrioventricular groovePCAT index. AIVGi= Anterior interventricular groovePCAT index.SIVGi= Superior interventricular groovePCAT index.IIVGi= Inferiorinterventricular groovePCAT index.GRS=Global radial strain. GCS=Global circumferential strain. GLS=Global longitudinal strain.

**Supplemental Table 6B.** Intra-observer variability for CMR indices and PCAT indices.

| **Variable** | **Bland-Altman analysis** | | **Coefficient of variation** | **Intra-class correlation coefficient** |
| --- | --- | --- | --- | --- |
|  | **Bias** | **95% limits of agreement** |  |  |
| **LVEF (%)** | -1.932 | -4.399 - 0.535 | 0.095 | 0.917 |
| **LV end-diastolic volume index (ml/m²)** | -1.177 | -4.872 - 2.517 | 0.081 | 0.898 |
| **LV end-systolic volume index (ml/m²)** | 0.758 | -0.547 - 2.064 | 0.049 | 0.988 |
| **LV mass (g/m^2^)** | 1.965 | -0.703 - 4.633 | 0.069 | 0.880 |
| **RVEF (%)** | -0.643 | -2.839 - 1.553 | 0.104 | 0.972 |
| **RV end-diastolic volume index (ml/m²)** | -0.407 | -3.334 - 2.519 | 0.090 | 0.977 |
| **RV end-systolic volume index (ml/m²)** | 0.982 | -0.967 - 2.931 | 0.095 | 0.992 |
| **Infarct size (% of LV mass)** | -0.525 | -1.515 - 0.465 | 0.069 | 0.962 |
| **Edema (% of LV mass)** | 0.306 | -1.337 - 1.950 | 0.105 | 0.978 |
| **Myocardial salvage index (% of LV mass)** | -0.795 | -1.841 - 0.251 | 0.086 | 0.989 |
| **GRS (%)** | -0.153 | -1.178 - 0.871 | 0.098 | 0.934 |
| **GCS (%)** | 0.293 | -0.272 - 0.859 | 0.074 | 0.947 |
| **GLS (%)** | 0.189 | -0.0847 - 0.462 | 0.062 | 0.975 |
| **RAVGi (mm/m^2^)** | -0.151 | -0.338 - 0.035 | 0.047 | 0.958 |
| **AIVGi (mm/m^2^)** | 0.0253 | -0.159 - 0.211 | 0.101 | 0.899 |
| **LAVGi (mm/m^2^)** | -0.0620 | -0.279 - 0.155 | 0.075 | 0.938 |
| **SIVGi (mm/m^2^)** | 0.231 | -0.174- 0.635 | 0.100 | 0.946 |
| **IIVGi (mm/m^2^)** | 0.145 | -0.075 - 0.364 | 0.091 | 0.941 |

**Abbreviations.** PCAT=Pericoronaryepicardial adipose tissue. STEMI= STelevation myocardial Infarction. MACE=Major adverse cardiac events.LVEF=Left ventricular ejection fraction. RVEF=Right ventricular ejection fraction. RAVGi=Rightatrioventricular groovePCAT index. LAVGi=Left atrioventricular groovePCAT index. AIVGi= Anterior interventricular groovePCAT index.SIVGi= Superior interventricular groovePCAT index.IIVGi= Inferiorinterventricular groovePCAT index.GRS=Global radial strain. GCS=Global circumferential strain. GLS=Global longitudinal strain.


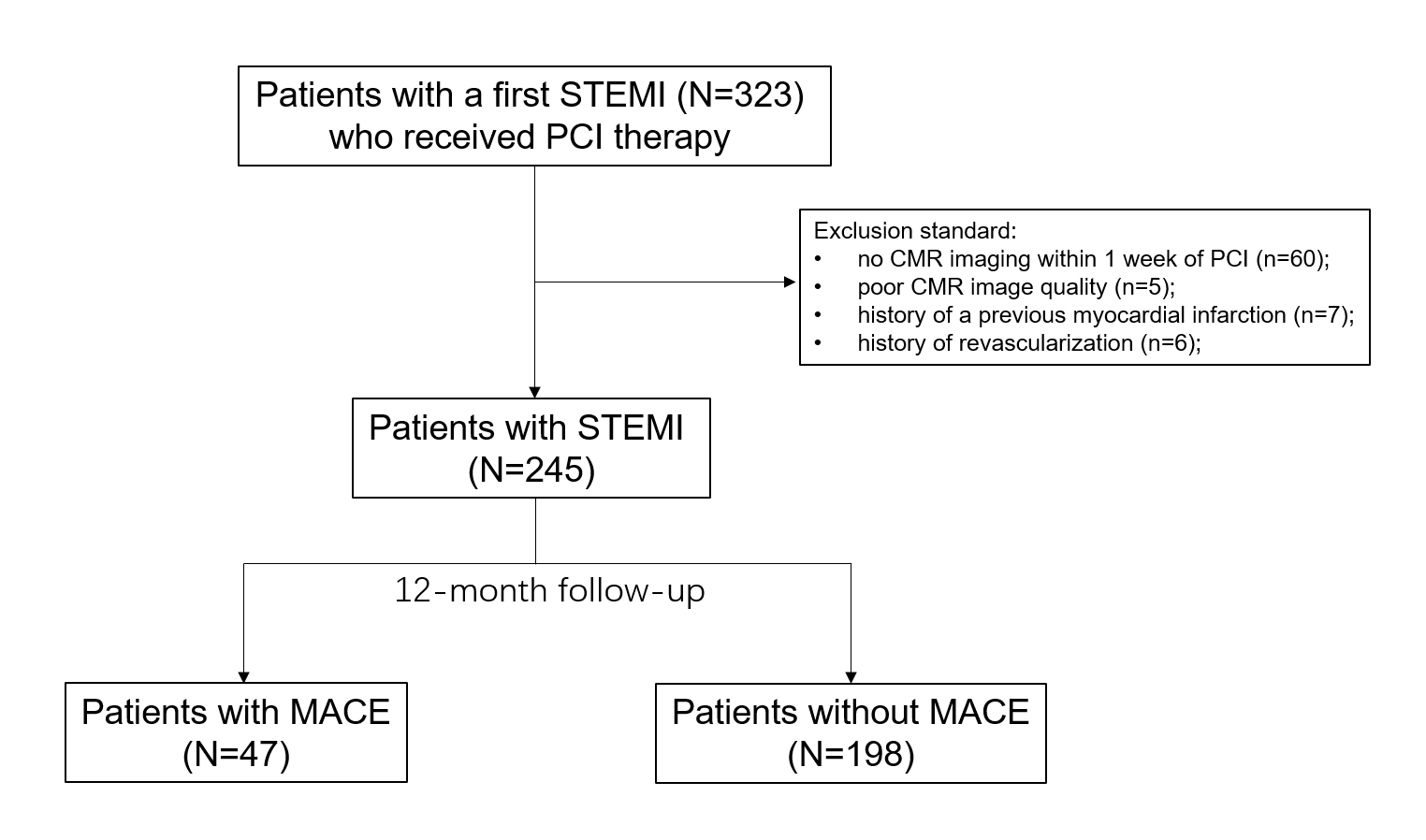


**Supplemental Figure 1.** Study flow chart. Of 323 screened patients, 245 were enrolled in the present study.


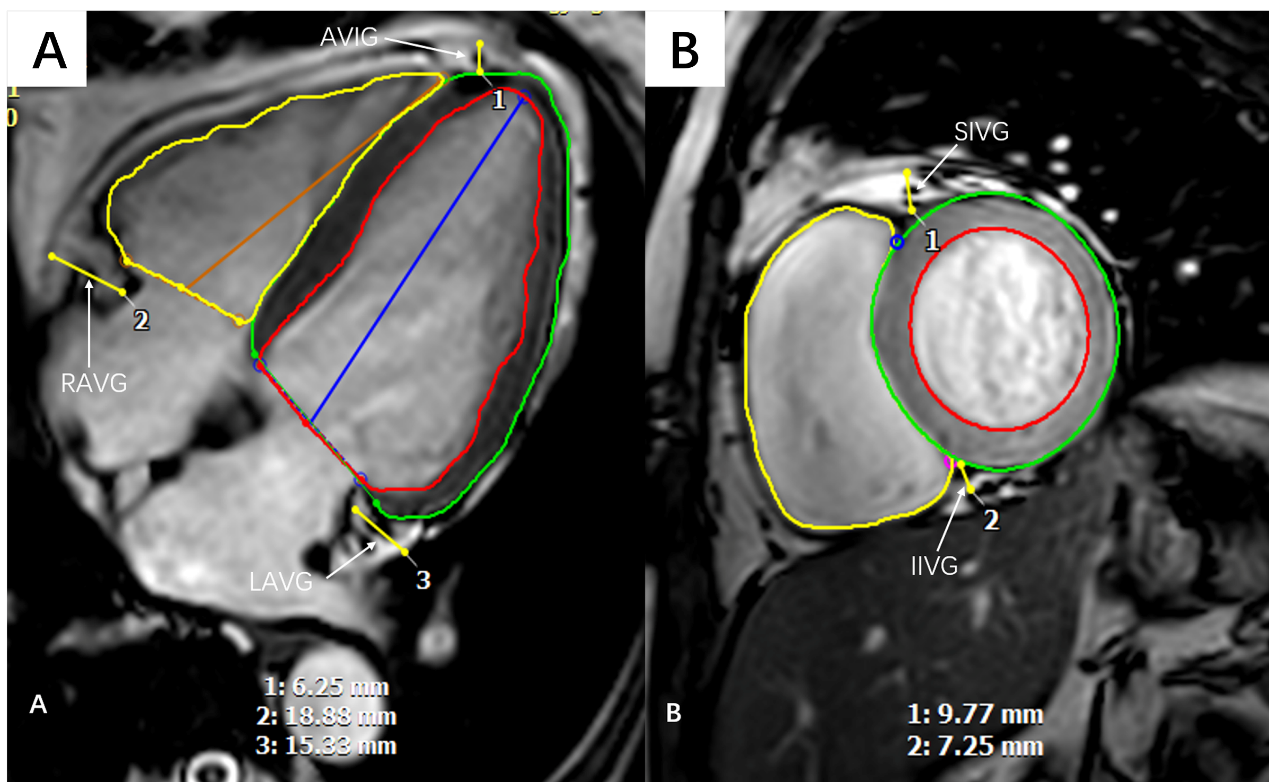


**Supplemental Figure 2.** PEAT thickness was measured on CMR cine images.The measurements were taken by two investigators blinded to all participant data. The PCAT of the right and left atrioventricular grooves (RAVG and LAVG, respectively) and the anterior interventricular groove (AIVG) were measured on the horizontal long-axis plane (a), whereas the PCAT of the superior and inferior interventricular grooves (SIVG and IIVG, respectively) were measured on the same basal parasternal short axis view just below the mitral valves (b).


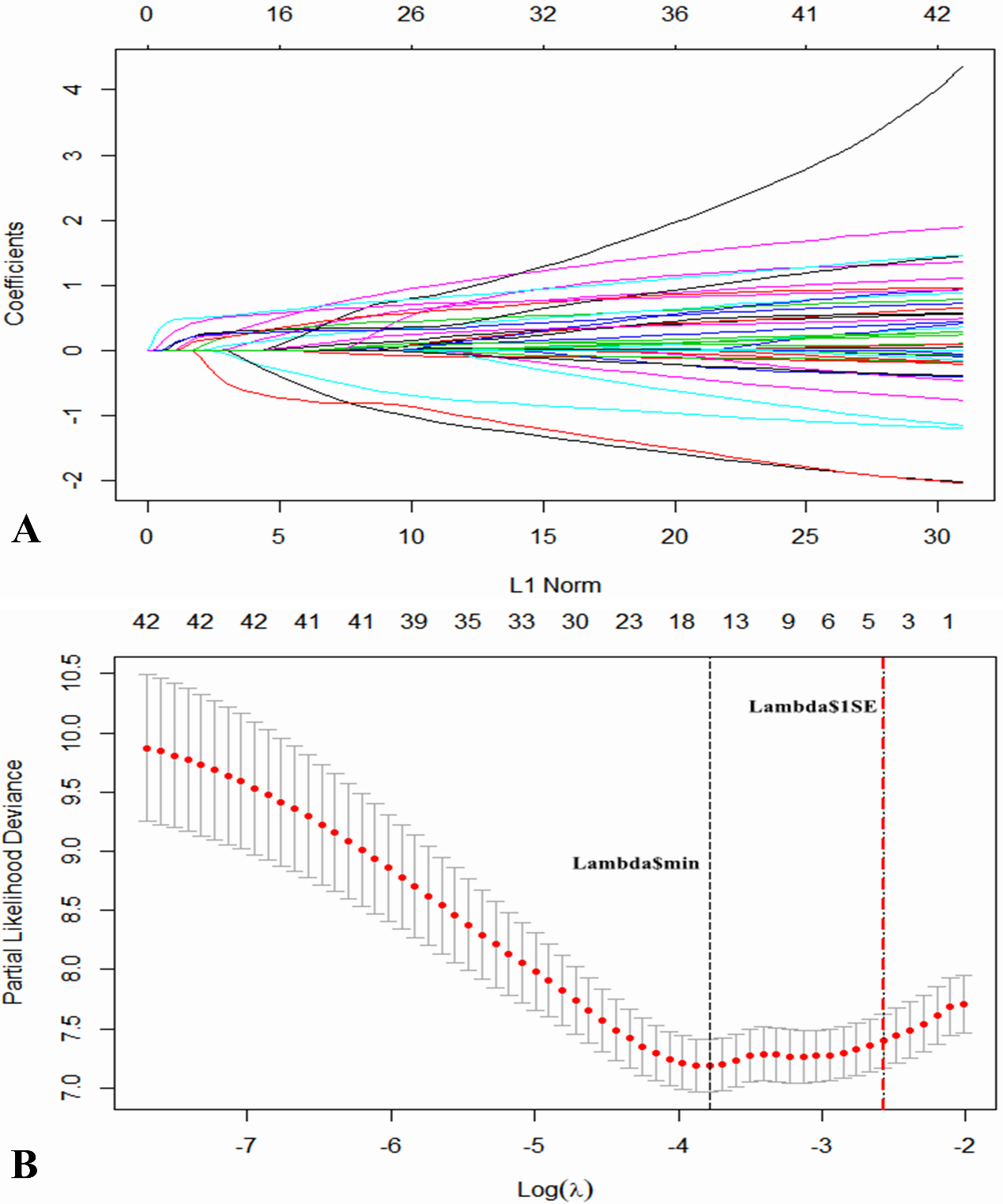


**Supplemental Figure 3.** Screening of predictor for risk of one-year MACE post STEMI using the LASSO (least absolute shrinkage and selection operator) Cox regression model. (A) LASSO coefficient profiles of all candidate variables except for PCAT thickness indexes. (B) The optimal parameters (λ) for the LASSO model were determined by tenfold cross-validation. *Notes: The black and red dotted vertical lines indicate the optimal λ values which were achieved using the minimum criteria and one standard error of the minimum criteria (1-SE criteria), respectively. Four variables (LVESVi, GRS, GCS and GLS) with non-zero coefficients were selected based on the 1-SE criteria.*

Abbreviations: GCS=Global circumferential strain. GLS=Global longitudinal strain. GRS=Global radial strain. LVESVi= left ventricular end-systolic volume index. MACE=Major adverse cardiac events. PCAT= Pericoronary adipose tissue. STEMI=ST elevation myocardial Infarction.


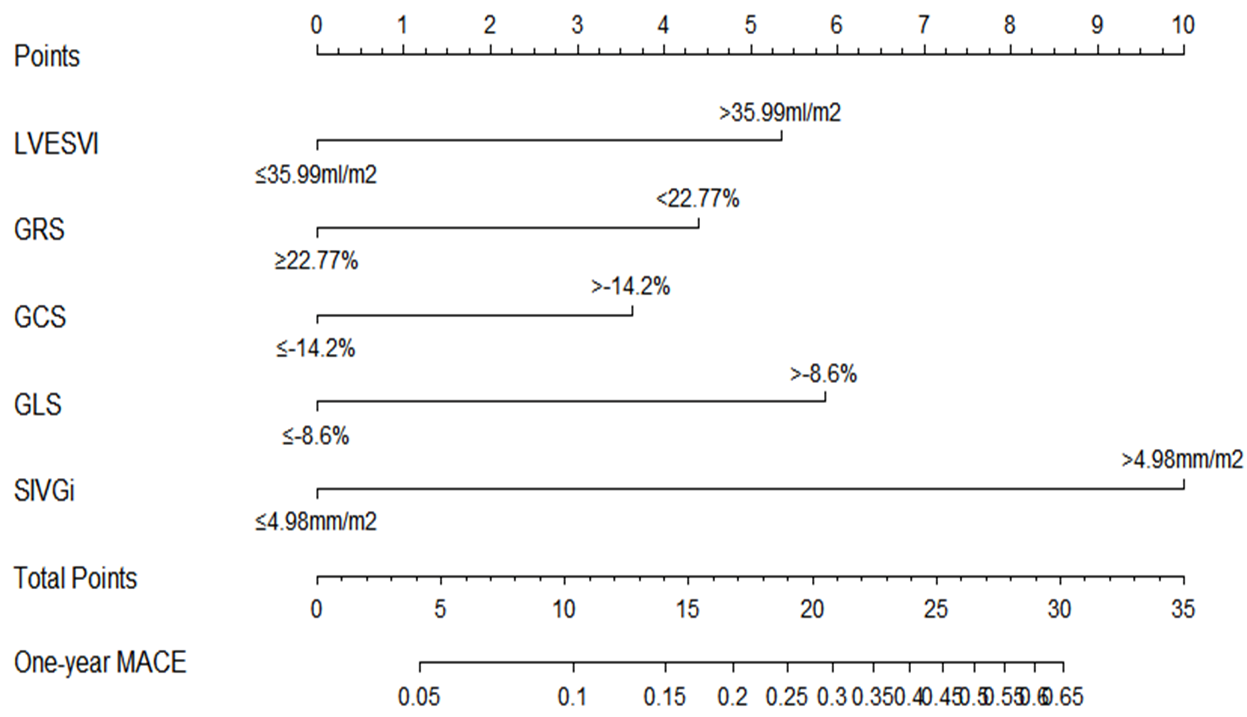


**Supplemental Figure 4.** The nomogram predicting the probability of one-year MACE.

To use the nomogram, an individual participant value is located on each variable axis, and a line is drawn upward to determine the number of points received for each variable value (LVESVi≤35.99ml/m^2^ = 0 points; LVESVi>35.99ml/m^2^ = 5.4; GRS≥22.77% = 0; GRS<22.77% = 4.4; GCS≤-14.2%= 0; GCS>-14.2%= 3.6; GLS≤-8.6%= 0; GLS>-8.6%= 5.8; SIVGi≤4.98mm/m^2^ = 0; and SIVGi>4.98mm/m^2^ = 10). The sum of these numbers is located on the Total Points axis to determine the risk of MACE. Abbreviations: GCS= Global circumferential strain; GLS=Global longitudinal strain. GRS= Global radial strain; LVESVi= left ventricular end-systolic volume index; MACE=Major adverse cardiac events; SIVGi= Superior interventricular groove PCAT thickness index.


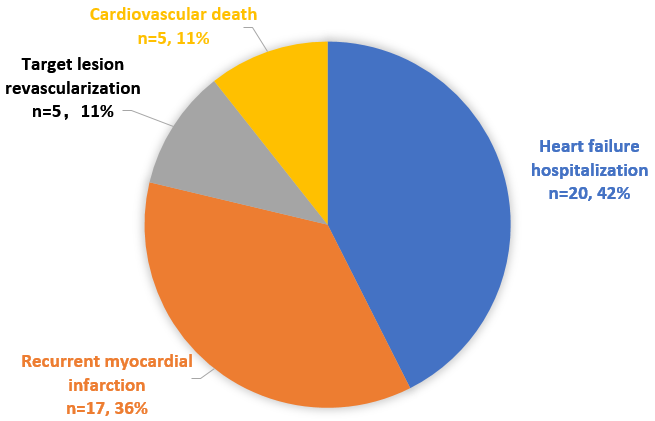


**Supplemental Figure 5.** The components of the 47 MACEs during follow-up.
